# Risk Factors and Predictive Model for Mortality of Hospitalized COVID-19 Elderly Patients from a Tertiary Care Hospital in Thailand

**DOI:** 10.3390/medicines10110059

**Published:** 2023-10-24

**Authors:** Mallika Chuansangeam, Bunyarat Srithan, Pattharawin Pattharanitima, Pawit Phadungsaksawasdi

**Affiliations:** 1 Department of Internal Medicine, Faculty of Medicine, Thammasat University Hospital, Thammasat University, Pathum Thani 12120, Thailand; 2 Division of Dermatology, Chulabhorn International College of Medicine, Thammasat University, Pathum Thani 12120, Thailand

**Keywords:** COVID-19, predictive model, elderly, mortality

## Abstract

**Background**: Early detection of elderly patients with COVID-19 who are at high risk of mortality is vital for appropriate clinical decisions. We aimed to evaluate the risk factors associated with all-cause in-hospital mortality among elderly patients with COVID-19. **Methods**: In this retrospective study, the medical records of elderly patients aged over 60 who were hospitalized with COVID-19 at Thammasat University Hospital from 1 July to 30 September 2021 were reviewed. Multivariate logistic regression was used to identify independent predictors of mortality. The sum of weighted integers was used as a total risk score for each patient. **Results**: In total, 138 medical records of patients were reviewed. Four identified variables based on the odds ratio (age, respiratory rate, glomerular filtration rate and history of stroke) were assigned a weighted integer and were developed to predict mortality risk in hospitalized elderly patients. The AUROC of the scoring system were 0.9415 (95% confidence interval, 0.9033–0.9716). The optimized scoring system was developed and a risk score over 213 was considered a cut-off point for high mortality risk. **Conclusions**: A simple predictive risk score provides an initial assessment of mortality risk at the time of admission with a high degree of accuracy among hospitalized elderly patients with COVID-19.

## 1. Introduction

SARS-CoV-2 coronavirus causes the infection called COVID-19, which originated from Wuhan city in China in December 2019 and subsequently spread worldwide. According to World Health Organization (WHO), there have been more than 700 million cases globally and almost 7 million people have died [1]. In South-East Asia, there have been more than 61 million confirmed cases and more than 800,000 patient deaths. More than three years since the first case was reported, COVID-19 is still killing, and it continues to mutate. There have been new variants emerging that cause new cases and deaths.

SARS-CoV-2 coronavirus is transmitted by exposure to infected secretions, which are mainly respiratory droplets and aerosols from person to person [2]. COVID-19 affects the respiratory system and can cause a variety of symptoms ranging from asymptomatic, mild (fever, sore throat, dry cough, and malaise), moderate (pneumonia without hypoxemia), to severe (multiorgan failure). COVID-19 can lead to severe pneumonia, respiratory failure, systemic inflammation, and even death. Older people have the highest health impact, which may result from low immunity and pre-existing co-morbidities. This is because of the atypical presentation of COVID-19 infection leading to an increased risk of misdiagnosis and inappropriate treatment [3]. Throughout the COVID-19 pandemic, older United States adults have been at an increased risk for COVID-19 associated illness and death, with older people accounting for more than 79% of deaths [4]. 

Older adults experience physiologic changes in their respiratory system, including decreased elastic recoil of the lung, reduced vital capacity and decreased mucociliary clearance leading to an ineffective cough and decreased pulmonary reserve. Immune dysregulation can decrease cell-mediated immune response and decreases antibody titer to specific foreign antigens, resulting in lower vaccine efficiency compared to young adults. In addition, older adults may have inflammation which can cause elevated serum levels of pro-inflammatory cytokines, including interleukin-1, interleukin-6, tumor necrotic factor (TNF), and C-reactive protein [5]. Therefore, when elderly patients contract COVID-19 infection, it might lead to a cytokine storm, which affects systemic inflammation, organ failure, and increases mortality [6].

Recently, the WHO declared an end to the COVID-19 global health emergency on 5 May 2023, marking a significant milestone in the global response to the pandemic. This development was largely attributed to the widespread administration of COVID-19 vaccines, which led to a dramatic reduction in both the severity of cases and hospitalization rates. However, new infections and reinfections continue to be reported, particularly among the elderly population. The risk factors and predictive models for mortality, which were crucial during the height of the pandemic, may remain relevant in certain patient populations.

Many studies suggest that risk factors such as old age [7,8,9,10], male sex [11], frailty [12], functional status [12,13], co-morbidities [14,15], and dementia [7,16,17] impact morbidity and mortality rate. Early detection of elderly patients with COVID-19 who are at high risk of mortality is of vital importance for appropriate clinical decisions. Although many risk score systems have been proposed [17], there is currently a lack of a specific scoring system designed for elderly patients with COVID-19. The objective of this study is to identify risk factors of mortality and develop a risk score for predicting mortality at the time of admission of elderly patients hospitalized with COVID-19.

## 2. Materials and Methods 

### 2.1. Study Design and Participants 

In the retrospective cohort study, two physicians reviewed the medical records of COVID-19 patients aged more than 60 years old admitted at Thammasat University Hospital from 1 July to 30 September 2021, which was the peak of the delta variant outbreak in Thailand. We searched for ICD-10 indicated COVID-19 infection. Patients were included if they tested positive for SARS-CoV-2 on reverse-transcriptase polymerase chain reaction (RT-PCR) from nasopharyngeal swabs, were hospitalized in Thammasat University Hospital, and were aged over 60 years old. We excluded patients if they were admitted to a hospital for less than 24 h, referred from other hospitals, or were admitted with other problems before the diagnosis of COVID-19 infection. 

### 2.2. Outcomes 

The primary outcome of this study is to evaluate the risk factors associated with all-cause in-hospital mortality among elderly patients hospitalized with COVID-19. The secondary outcome is to develop a predicting risk score for assessing in-hospital mortality. 

### 2.3. Data Collection 

The information recorded included baseline characteristics before COVID-19 infection such as age, sex, previous medical history, co-morbidities (hypertension, diabetes mellitus, chronic kidney disease, etc.), current medications, functional status, and history of COVID vaccination. Co-morbidity severity was assessed by the Charlson comorbidity index (CCI) and nutrition status was assessed by the body weight and body mass index (BMI).

Patient’s vital signs and symptoms, including fever, cough, fatigue, anorexia, loss of taste, anosmia, diarrhea, red eye and confusion, at the date of admission, were collected. Laboratory results at the admission date, including complete blood count, blood urea nitrogen, creatinine, electrolyte, C-reactive protein and liver function test, were collected. 

All complications during admission from doctor’s and nurse’s note (sepsis, hospital-acquired infection, respiratory failure, shock, acute kidney injury, acute hepatic failure, cardiac arrhythmia, heart failure, bleeding, delirium, fall and death) were reviewed. We also recorded the length of hospital stay, ICU stay and mechanical ventilator use.

Specific treatment (i.e., antiviral drugs, corticosteroid and anti-coagulant) and supportive treatment (respiratory support, antibiotic, inotropic drug, and nutrition supplement) were recorded. Details of other procedures, such as central line insertion, hemodialysis, endoscopy, and operation, were also collected. Functional status at discharge and the place of stay after hospital discharge (home, nursing home, another hospital) were collected. 

### 2.4. Statistical Analysis

Baseline characteristic data were presented as mean (SD) or median (IQR) for continuous variables and analyzed by using the Wilcoxon rank sum test. Univariate and multivariate logistic regression analyses were used to identify independent predictors of mortality with a *p*-value < 0.05. The variables with a *p*-value of less than 0.05 from univariate analysis, as well as previously reported associated variables (body mass index, chronic lung disease, ACE inhibitor, anticoagulant), were included in the multivariate analysis. The sum of the weighted integer was used as a total risk score for each patient. The predictive capability was identified using the area under the curve of receiver operator characteristics (AUROC). AUROC is a value between 0 and 1, where higher values indicate better performance. An AUROC of 0.5 represents random chance, while an AUROC of 1.0 indicates perfect discrimination. STATA statistical software (version 17.0) was used for all analyses.

## 3. Results

### 3.1. Baseline Characteristics 

For the 138 medical records of patients that were reviewed over a 12-week study period, 109 (78%) patients were alive after hospital discharge and 29 (22%) patients died during their hospital stay. 

The mean age of the survivor group was 71.6 years, and 50 patients (49.6%) were male. In the death group, the mean ages were 77.6 years, and 16 patients (55.2%) were male. The co-morbidities severity score between groups was different by using Charlson comorbidity index, 3.6 scores in the survival group and 4.8 scores in the death group (*p* = 0.001). The most prevalent co-morbidities were hypertension (67%), dyslipidemia (51%) and diabetes mellitus (43%). Cerebrovascular disease was 28% in the death group and 5% in the survivor group. All demographic data and patient baseline characteristics are described in Table 1.

The most common clinical presentations of patients were cough, dyspnea and fatigue. The presence of dyspnea was higher in the death group (86.2%) compared with the survivor group (49.5%) (*p* < 0.001). Signs of severe disease at admission were high respiratory rate and low oxygen saturation. All other symptoms at COVID-19 presentation are described in Table 2.

The laboratory data that affected mortality (*p* < 0.001) were total lymphocyte count, total neutrophil count, aspartate transaminase (AST), blood urea nitrogen, and glomerular filtration rate (GFR). Furthermore, the mean of CRP in the survivor group was 39 and 106 in the death group (*p* < 0.001) (Table 2).

### 3.2. Treatment and Complication

Anti-viral drugs (Favipiravir and Remdesivir) were used in all patients. Antibiotics were indicated in 16% of the survivor group and 93% of the death group. Systemic glucocorticoid was used as an anti-inflammatory therapy in 81% of patients (78% in the survivor group versus 97% in the death group). Invasive mechanical ventilator was used in 41% in the death group, but none in the survivor group. Therefore, the use of an invasive mechanical ventilator is a good predictor for poor outcomes. Non-invasive mechanical ventilators were used in 83% of the death group and 13% of the survivor group. The percentage of patients who were admitted to the intensive care unit was 14%. 

Acute respiratory failure is the most common cause of death in COVID-19 patients. Hospital-acquired pneumonia, and ventilator-associated pneumonia were the most common complications and affected the mortality rate (6.4% in the survivor group and 69% in the death group). Data are shown in Table 3.

### 3.3. Risk Factors for Mortality

The multivariate logistic regression analysis showed that age (Odds Ratio (OR) 1.27, 95% confidence interval (CI) 1.06–1.51), respiratory rate (OR 1.40, 95% CI 1.11–1.76), glomerular filtration rate (OR 0.93, 95% CI 0.88–0.98), and ischemic stroke (OR 89.20, 95% CI 1.83–4339) were independent risk factors for in-hospital mortality (Table 4).

### 3.4. Predicting Risk Score for Assessing Mortality

The four independent risk factors (age, respiratory rate, glomerular filtration rate and history of stroke) from multivariate logistic regression analysis were assigned to a predicting model. The lowest coefficient was used as a denominator for the other risk factors’ coefficients, and the results were rounded to integers for the predicting score (Table 5). The linear equation for predicting the score was

(age × 2) + (stroke × 18) + (respiratory rate × 6) − glomerular filtration rate

**Figure 1:**
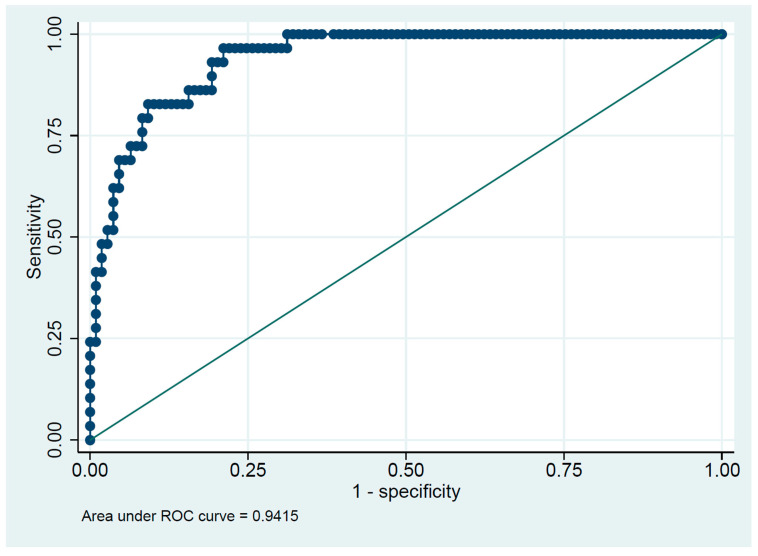
An area under receiver operating characteristic curve (AUROC) of predicting score using four variables (age, respiratory rate, GFR, and stroke) for predicting in-hospital mortality in elderly patients hospitalized with COVID-19.

Figure 1 shows the AUROC predicting score for predicting mortality of 0.9415 (95% confidence interval, 0.9033–0.9716). The mortality rate for patients with a predicting score of 234 or more was 82.8%, while the mortality rate was only 17.2% in patients with a lower predicting score. A predicting risk score of 234 was considered as a cut point for a high risk of death with a sensitivity and specificity of 82.76% and 90.83%, respectively.

## 4. Discussion

This study confirms the high mortality rate from COVID-19 among elderly patients, which is 22%. However, the mortality rate reported varies between countries due to several reasons, including differences in each government’s COVID-19 response policies. [9,18]. In Thailand in 2021, the government policy required that any COVID-19 patients be hospitalized, which resulted in a lower mortality rate compared to other countries. Within the elderly patient population, individuals of a more advanced age have higher mortality rates compared to their younger counterparts. Our study confirms that the mortality predictors for hospitalized COVID-19 patients include older age, co-morbidities (cerebrovascular disease and chronic kidney disease) and severity, signs of severe clinical presentation (confusion, dyspnea, fatigue, low oxygen saturation, low mean arterial pressure, high respiratory rate, and high pulse rate), and high inflammatory response (CRP, interleukin-6, total lymphocyte and total neutrophil count).

We found a significant association between a high-adjusted Charlson comorbidity index and a high mortality rate. This is consistent with Carilo-Garcia et al.’s study [12], which found an association between comorbidities, frailty, post-hospitalization functional deterioration, and mortality. Unlike the previous study [9,13], we did not find that male gender increases the risk for mortality. This might be a lack of statistical power of our sample in this result.

The most common clinical presentation is fever and cough, which is consistent with previous reports [8,9,11]. Ramos-Rincon JM et al.’s study [9] suggested severe COVID-19 at admission is related to poor prognosis, which is similar to our study. In addition, our study showed that the clinical presentations associated with a high mortality rate were high respiratory rate and low-mean arterial pressure.

The laboratory data that affected mortality was total lymphocyte count, total neutrophil count, AST, BUN, GFR and CRP. These results are consistent with the previous literature, which reported that the non-survivor group had higher white blood cell count and neutrophil to lymphocyte ratio, BUN, creatinine and CRP level [19]. These measurements can indicate a severe infection and potential end-organ failure.

Acute respiratory failure is the most common cause of death in COVID-19 patients in this study and worldwide [4,9]. Hospital-acquired pneumonia and ventilator-associated pneumonia were the most common complications and affected the mortality rate. Also, the invasive mechanical ventilator was used in 41% in the death group but none in the survivor group. So, using invasive mechanical suggest that patients have severe COVID-19 infection and are associated with high mortality rate. Only 14% of patients were admitted to the intensive care unit. 

Several prognostic scores for mortality in hospitalized patients have been proposed, for example, Acute Physiology and Chronic Health Evaluation (APACHE) II score, Modified early warning score (MEWS), Sequential Organ Failure Assessment (SOFA), and Clinical Frailty Scale (CFS). In Youg Sub Na et al.’s study [19], SOFA score showed the best performance in predicting the prognosis of elderly COVID-19 patients (AUROC 0.766, *p* < 0.001). This SOFA score assesses the performance of several organ systems in the body (neurologic, blood, liver, kidney, and hemodynamics).

Due to the slight differences in sensitivity and specificity between the full model incorporating all variables and the four-variable model, our study employed a calculated risk score based on four identified variables: age, respiratory rate, glomerular filtration rate, and history of stroke, which were selected based on their respective odds ratio. The optimized scoring system was developed, and a risk score over 213 was considered as a cutoff point for a high risk of death in this model. The sensitivity of this model is 82.76%, and its specificity is 90.83%. The prediction model from this study demonstrates an AUROC of 0.94, which is considered an excellent model for predicting mortality. We anticipate that the prediction model derived to this study can categorize elderly patients at a high risk of death. Compared to a previous study [18], our prediction model can be used for immediate identification after patients are admitted. However, validation of the models in other cohorts is recommended before use in clinical practice.

Our study has some limitations. Firstly, it is important to note that our study was conducted at a single center in Thailand over a short period of time, and had a relatively small sample size. Although we did not specifically identify the COVID-19 variant in each patient, our study coincided with the peak of the Delta variant outbreak in the region. We may assume most COVID-19 cases in our study were likely Delta variant infections. Secondly, due to the limitations in hospital capacity during the peak of the COVID-19 outbreak in Thailand, patients with more severe conditions or high co-morbidities were prioritized. This would have likely reduced the heterogeneity of the elderly patients included in this study. Thirdly, the availability of effective vaccines, anti-viral treatments and a new occurrence of a COVID-19 variant may impact the symptom and mortality rate, which should be considered when interpreting the results. Lastly, due to the global alleviation of the COVID-19 situation, we were unable to validate the risk score with a larger population.

## 5. Conclusions

In elderly patients hospitalized with COVID-19, a risk score using four identified variables at hospital admission—age, respiratory rate, glomerular filtration rate, and history of stroke—demonstrated good performance in predicting in-hospital mortality. This score could assist clinicians in identifying high-risk patients and in providing close monitoring to improve the prognosis of elderly populations in the context of COVID-19.

## Figures and Tables

**Table 1:** Demographic data and baseline characteristics of patients.

Characteristics	Total (*n* = 138)	Survive (*n* = 109)	Death (*n* = 29)	*p*-Value
Age (years), mean ± SD	72.8 ± 8.4	71.6 ± 7.6	77.6 ± 9.7	0.003
Male, n (%)	66 (48%)	50 (46%)	16 (55%)	0.41
Comorbidity, n (%)				
Diabetes mellitus	60 (43%)	44 (40%)	16 (55%)	0.21
Hypertension	93 (67%)	69 (63%)	24 (83%)	0.073
Dyslipidemia	71 (51%)	53 (49%)	18 (62%)	0.22
Coronary artery disease	17 (16%)	12 (11%)	5 (17%)	0.35
Congestive heart failure	2 (1%)	1 (1%)	1 (3%)	0.38
Ischemic stroke	11 (8%)	5 (5%)	6 (21%)	0.01
Hemorrhagic stroke	2 (1%)	0	2 (7%)	0.04
Connective tissue disease	1 (1%)	1 (1%)	0	1
Chronic kidney disease	11 (8%)	5 (5%)	6 (21%)	0.01
Cirrhosis	2 (1%)	1 (1%)	1 (3%)	0.38
Chronic lung disease	8 (6%)	7 (6%)	1 (3%)	1
Dementia	6 (4%)	3 (3%)	3 (10%)	0.11
Depression	1 (1%)	1 (1%)	0	1
Hearing problem	2 (1%)	1 (1%)	1 (3%)	0.38
Adjusted Charlson comorbidity index, mean ± SD	3.9 ± 1.8	3.6 ± 1.7	4.8 ± 2.1	0.001
Medication, n (%)				
Aspirin	36 (27%)	26 (25%)	10 (36%)	0.24
ACE inhibitors	22 (16%)	18 (17%)	4 (14%)	1
ARBs	23 (17%)	17 (16%)	6 (21%)	0.58
Corticosteroid	10 (7%)	7 (7%)	3 (11%)	0.43
Statin	67 (50%)	49 (46%)	18 (64%)	0.09
Metformin	35 (26%)	24 (22%)	11 (41%)	0.08
COVID Vaccine, n (%)				
None	39 (28%)	32 (29%)	7 (24%)	0.255
1 dose	90 (65%)	68 (62%)	22 (76%)	
2 doses	9 (7%)	9 (8%)	0	
COVID vaccine type, n (%)				
1st dose				
CoronaVac	7 (7%)	7 (9%)	0	
BBIBP-CorV	2 (2%)	1 (1%)	1 (5%)	0.132
ChAdOx1-S/AZD1222	34 (34%)	29 (38%)	5 (23%)	
2nd dose				
CoronaVac	1 (1%)	1 (1%)	0	
BBIBP-CorV	1 (1%)	1 (1%)	0	1.00
ChAdOx1-S/AZD1222	6 (4%)	6 (6%)	0	
BNT162b2	1 (1%)	1 (1%)	0	

**Table 2:** Clinical presentation and laboratory findings of patients.

	Total (*n* = 138)	Survive (*n* = 109)	Death (*n* = 29)	*p*-Value
Clinical presentation, n (%)				
Fever	101 (73%)	79 (72)	22 (76)	0.82
Cough	121 (88%)	94 (86)	27 (93)	0.53
Dyspnea	79 (57%)	54 (50)	25 (86)	<0.001
Fatigue	87 (63%)	62 (57)	25 (86)	0.004
Anorexia	34 (25%)	26 (24)	8 (28)	0.81
Anosmia	16 (12%)	15 (14)	1 (3)	0.19
Ageusia	18 (13%)	15 (14)	3 (10)	0.76
Diarrhea	19 (14%)	15 (14)	4 (14)	1
Confusion	6 (4%)	1 (1)	5 (17)	0.002
Physical examination, mean ± SD				
Body weight (kg)	63.1 ± 14	62.9 ± 15	64.2 ± 13	0.36
BMI (kg/m^2^)	24.7 ± 5.1	24.8 ± 5.3	24.1 ± 4.3	0.73
Body temperature (°C)	37.2 ± 0.9	37.1 ± 0.7	37.5 ± 1.2	0.34
MAP (mmHg)	96 ± 15	98 ± 12.8	90 ± 69	0.02
Pulse rate (/min)	88 ± 17	86 ± 16	94 ± 18	0.02
Respiratory rate (/min)	22 ± 5	20 ± 3	27 ± 7	<0.001
Oxygen saturation (%)	94 ± 9	96 ± 3	86 ± 16	<0.001
Laboratory, mean ± SD				
Hemoglobin (g/dL)	12 ± 2	12 ± 2	11 ± 2	0.003
Hematocrit (%)	37 ± 5	38 ± 5	34 ± 7	<0.001
WBC (/µL)	6302 ± 2929	5885 ± 2432	7869 ± 3994	0.004
TLC (/µL)	1494 ± 843	1629 ± 853	988 ± 578	<0.001
TNC (/µL)	4105 ± 2824	3530 ± 2175	6265 ± 3829	<0.001
Total protein (g/dL)	7.3 ± 0.7	7.4 ± 0.7	7.1 ± 0.7	0.045
Albumin (g/dL)	3.6 ± 0.4	3.7 ± 0.4	3.3 ± 0.4	<0.001
AST (U/L)	71 ± 201	48 ± 92	159 ± 393	<0.001
ALT (U/L)	44 ± 120	31 ± 35	91 ± 251	0.02
ALP (U/L)	83 ± 56	81 ± 57	91 ± 50	0.2
CRP (mg/dL)	54 ± 58	40 ± 47	106 ± 69	<0.001
BUN (mg/dL)	19 ± 12	16 ± 7	31 ± 19	<0.001
Creatinine (mg/dL)	1.2 ± 1.1	1.0 ± 0.7	2.1 ± 1.8	<0.001
eGFR (mL/min/1.73 m^2^)	65 ± 24	71 ± 21	43 ± 24	<0.001
Sodium (mEq/L)	136 ± 5	137 ± 5	133 ± 5	<0.001
Potassium (mEq/L)	3.8 ± 0.6	3.7 ± 0.5	4.2 ± 0.9	0.003
Chloride (mEq/L)	101 ± 5	101 ± 5	100 ± 6	0.17
Bicarbonate (mEq/L)	25 ± 3	26 ± 3	22 ± 4	<0.001

Body mass index—BMI, Mean arterial pressure—MAP, White Blood Cell—WBC, Total lymphocyte count—TLC, Total neutrophil count—TNC, Aspartate transaminase—AST, Alanine transaminase—ALT, Alkaline phosphatase—ALP, C-reactive protein—CRP, blood urea nitrogen—BUN, estimated glomerular filtration rate—eGFR.

**Table 3:** Treatment and complications of elderly patients with COVID-19 infection.

	Total (*n* = 138)	Survive (*n* = 109)	Death (*n* = 29)
Treatment, n (%)			
Anti-coagulant	70 (50%)	48 (44%)	22 (75%)
Antibiotic	44 (32%)	17 (16%)	27 (93%)
Steroid	113 (81%)	85 (78%)	28 (97%)
Inotropic drug	15 (11%)	1 (1%)	14 (48%)
Invasive mechanical ventilator	12 (9%)	0	12 (41%)
Non-invasive mechanical ventilator	38 (27%)	14 (13%)	24 (83%)
Intensive care unit	20 (14%)	5 (5%)	15 (52%)
Complication, n (%)			
Sepsis	23 (16%)	4 (4%)	19 (66%)
Respiratory failure	38 (28%)	9 (8%)	29 (100%)
Hospital-acquired pneumonia	27 (20%)	7 (6%)	20 (69%)
Renal failure	25 (18%)	8 (7%)	17 (59%)
Arrhythmia	9 (7%)	1 (1%)	8 (28%)
Pulmonary embolism	3 (2%)	0	3 (10%)
Delirium	16 (12%)	6 (6%)	10 (34%)

**Table 4:** Univariate and multivariate logistic regression analysis for mortality.

	Univariate Analysis			Multivariate Analysis		
Variable	Odds Ratio	95% CI	*p*-Value	Odds Ratio	95% CI	*p*-Value
Age	1.09	1.04–1.15	0.001	1.27	1.06–1.51	0.009
Female	0.69	0.30–1.57	0.374			
Under-weight ^1^	1.78	0.40–7.84	0.446	24.45	0.14–4312	0.226
Obesity ^2^	1.13	0.48–2.66	0.789	14.75	0.61–345	0.097
Diabetes mellitus	1.82	0.80–4.15	0.156	1.95	0.15–26.12	0.613
Charlson comorbidities index	1.38	1.11–1.71	0.004	0.41	0.16–1.09	0.074
Body temperature	1.53	0.98–2.39	0.063			
Pulse rate	1.03	1.01–1.06	0.019	1.06	0.98–1.15	0.136
Mean arterial pressure	0.96	0.93–0.99	0.012	0.99	0.89–1.10	0.902
Respiratory rate	1.37	1.20–1.55	<0.001	1.40	1.11–1.76	0.005
Hemoglobin	0.67	0.53–0.85	0.001	0.66	0.36–1.20	0.168
Total lymphocyte count	1.00	0.997–0.999	<0.001	1.00	0.99–1.00	0.125
Total Neutrophil count	1.00	1.0002–1.0005	<0.001	1.00	1.00–1.00	0.624
Albumin	0.13	0.05–0.37	<0.001	0.07	0.00–3.29	0.177
CRP	1.02	1.01–1.03	<0.001	1.01	0.99–1.03	0.532
Sodium	0.89	0.81–0.97	0.007	0.90	0.76–1.07	0.231
GFR	0.95	0.93–0.97	<0.001	0.93	0.88–0.98	0.01
Congestive heart failure	3.86	0.23–63.61	0.345			
Stroke	5.43	1.52–19.32	0.009	89.20	1.83–4339	0.023
Hypertension	2.78	0.98–7.87	0.054			
Chronic lung disease	0.52	0.06–4.41	0.549	0.05	0.00–98.67	0.442
ACE-inhibitor	1.32	0.76–2.29	0.325	1.83	0.11–29.53	0.672
Anti-coagulant	0.80	0.25–2.58	0.704	4.17	0.13–137	0.423
Statin	2.13	0.90–5.04	0.085			
Metformin	2.38	0.97–5.80	0.057			
Any COVID vaccine	1.31	0.51–3.36	0.580			

^1^ Under-weight was defined as BMI of less than 18.5 kg/m^2^. ^2^ Obesity was defined as BMI of 25 kg/m^2^ or more.

**Table 5:** The multiple correlation coefficients of the independent risk factors for mortality.

Variable	Coefficient	95% CI	*p*-Value
Age	0.10	0.02–0.17	0.01
Respiratory rate	0.32	0.16–0.49	<0.001
GFR	−0.05	−0.09 to −0.02	0.001
Stroke	0.93	−0.775 to 2.634	0.285

## Data Availability

Data are available from the corresponding authors upon reasonable request.
